# Challenges and Solutions to Maintaining Health Science Education in Gaza Since October 2023: An Expert Perspective

**DOI:** 10.1002/hsr2.73050

**Published:** 2026-08-13

**Authors:** Suad J. Ghaben, Charlotte Chatto, Ihab A. Naser, Abdelraouf A. Elmanama

**Affiliations:** ^1^ Department of Physical Therapy, Faculty of Applied Medical Sciences Al‐Azhar University‐Gaza Gaza Strip Palestine; ^2^ University of the Cumberlands Williamsburg Kentucky USA; ^3^ Department of Clinical Nutrition, Faculty of Applied Medical Sciences Al‐Azhar University‐Gaza Gaza Strip Palestine; ^4^ Medical Laboratory Sciences Department, Faculty of Health Sciences Islamic University of Gaza Gaza Strip Palestine

**Keywords:** Gaza Strip, health science education, online learning, resilience, telerehabilitation

## Abstract

**Background and Aims:**

Relaunching Health Science Education (HSE) in the Gaza Strip posed a significant challenge following the large‐scale attacks since October 2023. We aim to explore the institutional experiences in relaunching HSE during wartime, with emphasis on the solutions implemented and policy recommendations that emerged.

**Methods:**

This perspective draws on institutional records, published damage and casualty reports, and the authors' direct observations as faculty and administrators, covering developments from October 2023 to January 2026, to analyze the process through which HSE was relaunched in the Gaza Strip.

**Results:**

The two major universities offering HSE in Gaza sustained varying degrees of damage, leading to distinct response strategies. Al‐Azhar University in Gaza prioritized restoring independent digital infrastructure and establishing international clinical placements. The Islamic University of Gaza emphasized community‐based recovery, symbolizing resilience and continuity. Both institutions implemented academic flexibility and strategic partnerships and adopted the philosophy of resilience.

**Conclusion:**

Despite the progress achieved in relaunching HSE via online learning and resuming clinical training, further policy measures are required to strengthen HSE in the Gaza Strip.

## 1 Background

Since October 7, 2023, the delivery of higher education (HE) in Gaza has been almost completely suspended due to widespread infrastructure destruction, displacement, resource shortages, communication breakdowns, and the killing, injury, and detention of staff and students [1, 2]. Health Science Education (HSE), including clinical training, was also halted across all facilities [3]. Given each discipline's required professional competencies and complex clinical arrangements [4, 5], HSE faces even greater barriers to resumption during the war [3].

By October 2023, 11 institutions offered HSE in Gaza, with Al‐Azhar University‐Gaza (AUG) and the Islamic University of Gaza (IUG) serving as the primary providers. Together, they host faculties of Medicine, Nursing, Pharmacy, Dentistry, and Applied Medical Sciences, supplying approximately 80% of healthcare professionals (HCPs) [6, 7]. These institutions, along with others, were repeatedly targeted, resulting in the demolition of 63 buildings, including all campuses in Almoghraqa Area, and severe damage to facilities in Gaza City and Khan Younis [8]. Remaining structures were repurposed to shelter internally displaced persons. The loss of human lives on these campuses has been significant. By November 2025, 241 staff and 1351 students were killed, with thousands more injured [8]. Clinical training facilities, including hospitals and clinics, were also destroyed, making training impossible; moreover, universities' financial constraints further limited the ability to sustain clinical training. From the United Nations Human Rights report in July 2025, 1581 HCPs have been killed [9], underscoring the urgent need to resume HSE and clinical training to replenish the healthcare workforce. This perspective draws on institutional experiences, administrative records, and the authors' observations rather than on empirical data collection. It identifies how two major universities in Gaza sustained health HSE during active conflict and proposes policy‐relevant recommendations.

## 2 Approach

**FIGURE 1:**
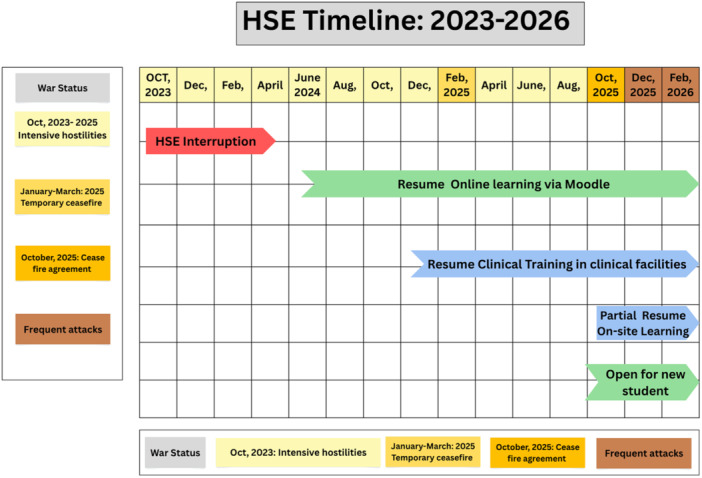
Timeline of health science education (HSE) resumption at Al‐Azhar University‐Gaza (AUG) and the Islamic University of Gaza (IUG) relative to the war status, October 2023–October 2025 until January 2026. The timeline maps war‐status phases (active conflict, partial ceasefire, and full ceasefire) against key HSE milestones (suspension, resumption of online learning via the Moodle Learning Management System, resumption of clinical training, and reopening to new students). HSE, health science education. Elaborated by the authors.

This Perspective is grounded in the authors' institutional roles at AUG and IUG and their direct involvement in coordinating the emergency response described here. It draws on three source types: (a) internal administrative and enrollment records maintained by AUG's Admissions, and Registration offices, and comparable IUG institutional communications; (b) situation reports and damage assessments published by international bodies, including the United Nations Office of the High Commissioner for Human Rights (OHCHR), UNESCO, and the Institute for Palestine Studies; and (c) the authors' direct observations as faculty and administrators engaged in the response. This Perspective, as an expert opinion rather than an empirical study, covers institutional developments from October 2023 through January 2026, spanning the period before and after the October 2025 ceasefire, as depicted in Figure 1.

Existing knowledge on higher education in Gaza during the war has documented broad patterns of infrastructure destruction and educational disruption affecting universities generally [1, 2], yet the distinct, licensure‐driven demands of health science education, which require accredited clinical placements, laboratory access, and Ministry of Health‐regulated competency verification, remain largely unexamined. This gap is consequential: without functioning HSE pathways, the replenishment of Gaza's healthcare workforce cannot keep pace with wartime attrition and the population's rising health needs. This Perspective addresses that gap by documenting how AUG and IUG sustained HSE delivery amid active conflict and translating those institutional experiences into policy‐relevant recommendations.

## 3 Relaunching Health Science Education (HSE)

AUG and IUG, Gaza's largest universities, which supply most of the country's healthcare professionals, faced differing levels of destruction and response. Figure 1 illustrates the timeline of HSE in Gaza in relation to the war status. HSE was suspended for 8 months following the war's onset, with online learning reintroduced via the Moodle platform in June 2024, and clinical training resumed in the subsequent semester. After the October 2025 ceasefire, universities reopened to new students and partially resumed on‑site education. Continued attacks and a blockade, however, have hindered full restoration, underscoring the need for sustained and intensified efforts to sustain HSE.

AUG and IUG, Gaza's largest universities, which supply most of the healthcare professionals (HCPs), faced varying levels of destruction and responses. The following sections describe how each university was impacted and its response strategies, reflecting the specific needs it must address. For example, AUG focused on restoring independent digital infrastructure and establishing international clinical placements. IUG concentrated its efforts on community‐based restoration, symbolizing a dedicated presence. With a philosophy of resilience, they both implemented academic flexibility and strategic partnerships. Table 1 summarizes these institutional differences across five dimensions: extent of damage, primary strategy, key enabling partners, measurable outcomes, and residual gaps.

**TABLE 1:** Comparative summary of institutional damage, strategy, partners, outcomes, and residual gaps: AUG vs. IUG.

Dimension	AUG	IUG
Extent of damage	‐ Partial destruction of buildings. ‐ Full Almoghraqa campus loss; ‐ severe damage to the campus in Gaza City.	‐ ~90% of buildings destroyed; ‐ digital system collapse; − 74 faculty and ~500 students killed*
Primary strategy	‐ Independent digital infrastructure (Moodle, hosted by Al‐Balqa Applied University)‐International clinical placement network	Phased restoration of physical/virtual spaces; ‐ community and diaspora engagement; ‐ academic flexibility (deadline extensions, fee waivers)
Key enabling partners	‐ West Bank universities, ‐ Palestinian Ministry of Higher Education (MoHE), ‐ PalMed, NAAMA, ‐ Taawon,	‐ Community engagement ‐ Diaspora academics; ‐ international networks; ‐ Turkish–Palestinian Friendship Hospital
Measurable outcome	‐ First Gaza university to resume online learning (May 2024); − 9000 students at relaunch; ‐ 14,000 new students in 2025; 27,000 total enrollment*	‐ Resumed limited on‐site instruction after the October 2025 ceasefire, ‐ Leveraging pre‐existing e‐learning infrastructure* ‐ The third among the Palestinian universities on the 2026 QS rankings.
Residual gaps	‐ Full on‐site and lab‐based instruction not yet restored; ‐ Continued dependence on externally hosted digital infrastructure.	‐ Digital and research infrastructure largely unrestored; ‐ No independent LMS relaunch reported to date*

* Institutional records and reports.

Abbreviations: AUG, Al‐Azhar University‐Gaza; IUG, Islamic University of Gaza; LMS, learning management system; MoHE, Ministry of Higher Education.

**FIGURE 2:**
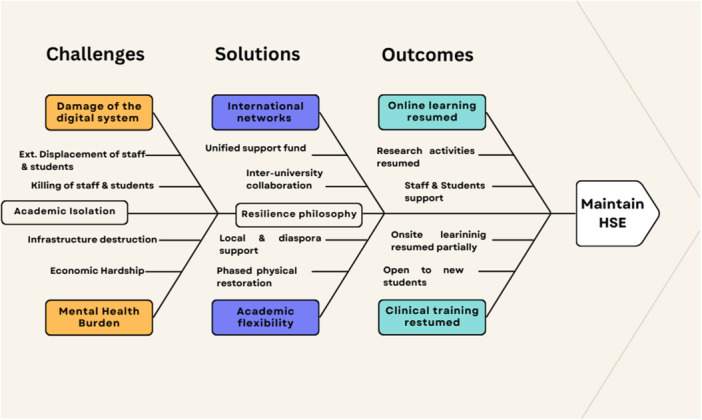
War‐induced challenges, institutional solutions, and outcomes affecting health science education (HSE) in Gaza since October 2023. The figure presents a three‐column framework mapping (left) war‐induced challenges (infrastructure and human capital loss, financial hardship, psychological burden, governance gaps, and international isolation) to (center) the response strategies adopted by Al‐Azhar University‐Gaza (AUG) and the Islamic University of Gaza (IUG), and (right) the resulting institutional outcomes (resumption of online and clinical learning, partial resumption of on‐site education, and sustained enrollment). Elaborated by authors.

Despite the unprecedented devastation since October 2023 and the variation between the two cases presented here, HSE was maintained in Gaza. Figure 2 illustrates the war‐induced challenges, the solutions implemented, and the resulting outcomes of their efforts to maintain HSE. This three‐column framework can guide the maintenance of HSE under active conflict conditions.

### 3.1 Al‐Azhar University‐Gaza (AUG)

Before October 2023, AUG successfully positioned itself as a leading academic hub through infrastructure investment, research excellence, international collaboration, and market‐aligned education. However, the infrastructure damage and the massive collapse of digital infrastructure and databases in the aftermath of the October 2023 events had paralyzed the system, requiring the suspension of HSE Education delivery.

### 3.2 Emergency Transition, Digital Autonomy, and Resilience

AUG addressed both physical and digital infrastructure damage through an emergency plan supported by interuniversity collaborations among West Bank universities, facilitated by the Palestinian Ministry of Higher Education. Resources for digital communication and for staff who volunteered from West Bank universities enabled secure education at AUG, although curriculum and logistical challenges persisted. By May 2024, the AUG was the first Gaza university to resume education and independently relaunched its distance learning via Moodle, hosted by Al‐Balqa Applied University. Enrolling over 9000 students (Institutional enrollment records), demonstrating institutional resilience and sustained student engagement. The university staff, including those abroad, volunteered to sustain teaching and covered the workloads of colleagues unable to teach due to the conflict. With guidance and oversight from the AUG University Council, quality standards were enforced while allowing flexibility in the process. Student feedback and performance on technical evaluations informed refinements for subsequent semesters, demonstrating not only resilience but development of the critical skills of digital autonomy.

### 3.3 Globalized Clinical Training (Clinical Diplomacy)

To maintain HSE, during the suspension of clinical training, the AUG implemented a multi‐phased strategy. First, theoretical courses were provided digitally through Moodle, while the practical and clinical components were deferred to subsequent semesters. Later, international partners provided virtual simulations, pre‐recorded faculty demonstrations, and open‐source medical resources to supplement instruction. Second, students were integrated into healthcare placements, locally and globally. AUG coordinated an international clinical training network, leveraging partnerships with PalMed and NAAMA, to secure clinical placements abroad for the externally displaced students, who were successfully integrated into medical and dental programs in South Africa, Malaysia, Pakistan, Norway, Iraq, and Egypt. For the students remaining in Gaza, clinical training was adapted to the available, albeit strained, local health facilities. Ultimately, quality assurance was maintained through standardized curricula and performance evaluation rubrics, with transparent reporting and direct communication. With careful oversight from the host institution, final‐year students and interns were enabled to graduate and address the increasingly critical workforce shortage of HCPs.

### 3.4 Financial Recovery and Institutional Survival

AUG secured institutional survival through global partnerships and strategic financial initiatives. The International Emergency Committee, managed by Taawon, a non‐profit civic organization, directed international donations to Gaza's universities, while a graduate Relief initiative cleared tuition debts for medical graduates, sustaining healthcare capacity and family livelihoods. Faculty research, initially disrupted, resumed with support from international affiliations, restoring scientific productivity.

Institutional resilience was further strengthened by faculty integration into prestigious universities across North America and Europe, fostering partnerships and research opportunities. Notably, the University of Alberta's 2025 remote research program enabled displaced Gaza scientists to continue their work, and several twinning and MoUs were signed with European universities. Current priorities include rebuilding essential health science laboratories and transitioning to comprehensive in‑person education, supported by prudent financial and administrative management.

### 3.5 “Nothing Is Impossible With the Palestinians” Philosophy

Notable outcomes of the AUG's strategies to resume education are the full resumption of online learning and clinical training, and partial resumption of on‐site learning. There was a marked increase in student enrollment, with over 14,000 new students joining the AUG in 2025, bringing total student enrollment to approximately 27,000 (Institutional enrollment records). These records reflect the Palestinians' resilience, eagerness to learn, and the community's trust in academic institutions, particularly the AUG, which created a tangible academic reality in a seemingly impossible environment, reinforcing the idea that “education” is a cornerstone of the community's future.

### 3.6 The Islamic University of Gaza (IUG)

The IUG is a premier Palestinian university ranked 99th in the QS Arab University Rankings 2026 and third among Palestinian universities [10], despite frequent and intensive attacks. Recognized for its unique infrastructure, community involvement, and research excellence, it is also partnered with the Turkish–Palestinian Friendship Hospital as an academic hospital. Due to the massive attacks on the IUG campuses since October 2023, 90% of buildings have been destroyed, causing the collapse of the digital system, killing 74 elite professors and 500 students (institutional report), and requiring the suspension of the delivery of education until June, 2024 [8].

### 3.7 The War Impacts and Educational Challenges

The IUG assets were intensively targeted since October 2023. Approximately 90% of IUG's buildings were destroyed, including classrooms, laboratories, libraries, and offices. The remaining structures were severely damaged and repurposed as shelters for displaced families, obstructing the reconstruction of the academic buildings. Human capital was also targeted, with dozens of faculty and staff and hundreds of students killed. The deaths of senior faculty disrupted teaching continuity, mentorship, and institutional memory. Digital and Research Infrastructure: servers, networks, and online platforms were destroyed, halting digital learning. Laboratories, equipment, and datasets were lost, terminating ongoing research and graduate projects.

The economic hardship and widespread poverty made tuition unaffordable and led to the collapse of institutional income. Salaries were delayed or left unpaid, lowering morale and prompting emigration, adding to the brain drain from the killing of the faculty and staff. Furthermore, the psychological trauma and mental burden, grief, and fear affected students and staff, undermining motivation and learning. Absence of mental health support exacerbated these challenges. The governance and policy constraints stemming from the lack of a national emergency plan created leadership gaps, disrupted accreditation, and hindered the development of disaster‐resilient educational strategies. The International Isolation exacerbated the situation, leading to the cessation of academic exchanges, partnerships, and access to journals and grants, leaving IUG disconnected from global academia and limiting recovery opportunities.

### 3.8 Resilience Strategies

#### 3.8.1 Adaptive Leadership and Institutional Commitment

University leaders framed the continuation of education as both a moral duty and a strategic necessity. Public messaging positioned education as “a lifeline for hope and resilience,” motivating staff and students to persist in their studies despite systemic collapse.

#### 3.8.2 Phased Restoration of Physical and Virtual Learning Spaces

IUG repaired partially damaged buildings for limited classroom use and established temporary facilities where necessary, prioritizing essential courses, particularly for graduating students.

#### 3.8.3 Academic Flexibility and Student Support

The university employed limited online instruction during campus closures and, after the ceasefire, adopted blended approaches combining in‑person teaching with digital instructional tools. Its long‑standing e‑learning system and pre‑recorded lectures, previously utilized during the COVID‑19 pandemic, facilitated continuity of learning. IUG adapted its regulations to wartime conditions by extending deadlines, relaxing attendance requirements, modifying assessments, waiving fees, and offering informal scholarships to prevent student attrition.

#### 3.8.4 Community and Diaspora Engagement

IUG leveraged local and international networks, with external academics providing remote teaching, mentoring, and curriculum support to offset staff shortages and sustain operations.

#### 3.8.5 “Education Is a Lifeline for Hope and Resilience” Philosophy

The partial resumption of campus activities at IUG served as a declaration of education's role in Gaza's future, restoring normalcy, morale, and institutional confidence. Despite severe destruction and systemic collapse, the university employed pragmatic and flexible strategies to sustain learning, and 6501 students (2805 males and 3693 females) were enrolled during 2025 (Institutional enrollment records). This case illustrates higher education as essential to social survival and recovery in conflict settings, offering policymakers clear evidence that protecting universities is an investment in peace, dignity, and reconstruction.

## 4 Solutions and Policy Recommendations

The mentioned HSE challenges during the war and the growing need for skilled HCPs necessitate creative solutions. The transition from face‐to‐face or a blended hybrid format to entirely online learning was necessary for all HSE in Gaza. Although such transitions were experienced during the COVID‐19 pandemic, the unprecedented lack of infrastructure and resources, along with the burden on physical and mental health, have made the HSE more challenging. The lessons learned and the digital platforms created or enhanced during the pandemic now offer the most promising opportunities for HSE in Gaza. To ensure the long‐term survival and growth of the HSE in Gaza and address the increasing challenges and demands, a set of solutions and policy recommendations should be considered. Of these, Moodle‐based online learning and video‐based skills capture are already operational at both AUG and IUG; the remaining tools discussed below, simulation labs, case‐based platforms, and telerehabilitation‐based clinical training, are prospective recommendations for further scale‐up rather than practices currently implemented for HSE at either institution. Policies target the long‐term restoration of on‐site learning, while solutions underscore the enhancement of online learning by integrating new, effective tools, such as simulation labs, to replace practical components [11, 12], case‐based education [13], and telerehabilitation (TR) to replace the unavailability of on‐site health services and clinical training [14, 15, 16].

Learning management systems such as Moodle [17] were already in place for access to asynchronous materials and assessments. Every effort must now be made to ensure that these materials are engaging and effective, incorporating high‐impact educational practices that are supported by the science of learning [18]. Traditional lectures and textbook readings could be augmented or even replaced with cloud‐based educational programs that incorporate games, spaced recall, and patient cases that require application of knowledge. In addition, assessments must be sufficiently rigorous and remotely proctored to verify students' knowledge.

Opportunities for foundational sciences such as human anatomy and human neuroanatomy that offer dissection videos, explainer videos, text, interactive images, and 3D models [11, 19, 20, 21]. Most are free or low‐cost for low‐ to middle‐income countries, or offer free faculty access. Platforms that offer interactive case studies and simulations, especially ones that vary and evolve in complexity throughout the curriculum, allow for faculty feedback, encourage the development of clinical reasoning, and require the application of foundational sciences [11, 13]. Students also require feedback as they develop their clinical skills and to confirm their mastery before actual patient care [22, 23]. Programs that allow students to upload videos from their phones, of themselves performing manual skills such as wheelchair transfers, or performing an interview with a simulated patient [12, 24] give faculty a mechanism to provide written or audio‐timestamped feedback and to assign grades when reviewing the video. When the student watches the video back, it simulates real‐time feedback.

Most HSE programs require both didactic and clinical training to qualify for licensure from the Palestine Ministry of Health. TR platforms allow practitioners to examine and treat patients virtually [14, 15, 16], and there is potential to harness their capabilities for student training as well. Students in any location can potentially work with an on‐site instructor at a clinic or hospital that they cannot travel to due to impassable roads or security concerns. Their instructor could even be in the field with non‐profit health organizations or with displaced patients living in tents, and the student could not only have a meaningful clinical experience, but also gain skills in TR for the future.

Realizing these recommendations depends on infrastructural requirements that remain only partially met in Gaza. Stable electricity, bandwidth, and device access, the same constraints that led AUG to rely on partner‐hosted servers rather than locally hosted platforms, will continue to determine which tools are feasible in the near term. We recommend prioritizing low‐bandwidth, asynchronous solutions (e.g., downloadable video and text‐based materials) before higher‐bandwidth, synchronous applications such as real‐time simulation or telerehabilitation become broadly viable, alongside continued investment in partner‐hosted infrastructure as a practical interim model.

To ensure long‐term survival and enhancement of HSE, robust governmental and institutional frameworks are essential. This necessitates a strategic pivot by the Ministry of Higher Education to secure funding and establish policies for the resumption of on‐site learning, while leveraging its legal and international standing to safeguard educational infrastructure. Despite the limitations of the international bodies in protecting human rights during the Gaza war, UNESCO remains a critical advocate. It is asked to utilize its legal mandate to advocate for institutional security and the Palestinians' right to education. Through a partnership with the Palestinian Ministry of Higher Education, UNESCO provides digital infrastructure and learning spaces, supports damage assessment, policy alignment, and learning continuity, and promotes safe academic environments through mental health initiatives and awareness programs [25, 26].

In alignment with the three pillars of higher education: teaching, research, and community service, universities in Gaza should transcend their traditional educational role. To effectively address urgent social challenges, universities should prioritize community integration, align the institutional role with the community's needs, conduct community‐aligned research to provide empirical solutions to localized crises, and integrate service learning to enhance students' problem‐solving skills and civic engagement.

## 5 Conclusion

Despite catastrophic infrastructure and human capital losses, AUG and IUG each sustained core HSE functions through complementary strategies: AUG through independent digital autonomy and globalized clinical placement, and IUG through community‐anchored, phased restoration, demonstrating that health professions education can continue, in adapted form, even under active conflict. Both institutions' progress in resuming online instruction, clinical training, and partial on‐site learning offers a proof of concept for conflict‐affected HSE elsewhere, but it remains fragile: on‐site laboratory and clinical infrastructure are still far from restored, and continued blockade and insecurity threaten these gains. Sustaining and scaling this progress requires coordinated action: expanded international clinical partnerships, low‐bandwidth‐first digital tools, dedicated Ministry of Higher Education funding and policy support, and advocacy by international bodies such as UNESCO for the protection of academic infrastructure, alongside the continued collaboration of policymakers, university leaders, staff, students, and the wider community. Although Gaza universities managed to resume online learning, clinical training, and partial on‐site learning, sustaining HSE remains challenging and necessitates the sustained collaborative efforts of international and local bodies, policymakers, university leaders, staff, students, and community engagement.

## Author Contributions

**Suad J. Ghaben:** conceptualization, writing – original draft, writing – review and editing, visualization, methodology, project administration, formal analysis, funding acquisition, data curation, resources. **Charlotte Chatto:** writing – original draft, writing – review and editing, methodology, formal analysis. **Ihab A. Naser:** writing – original draft, writing – review and editing, formal analysis, resources, methodology, conceptualization. **Abdelraouf A. Elmanama:** writing – original draft, writing – review and editing, resources, methodology, formal analysis, conceptualization.

## Funding

The authors have nothing to report.

## Disclosure

The main and corresponding author, Suad J. Ghaben, is the manuscript guarantor, had full access to all of the data in this study, and affirms that this manuscript is an honest, accurate, and transparent account of the perspective being reported; that no important aspects have been omitted; and that any discrepancies from the work as planned have been explained.

## Ethics Statement

This Perspective did not involve the collection of data from human participants and is not a human‐subjects study; consequently, ethics committee approval and informed consent were not required. No identifiable patient or student information is reported.

## Conflicts of Interest

The authors declare no conflicts of interest.

## Data Availability

This is an expert Perspective and does not report new primary research data. All figures and claims are derived either from the authors' institutional records (identified as such in the text) or from publicly available third‐party reports, each cited at the point of use; no additional dataset is associated with this article.
